# Public Health Interventions in the Emergency Department: A Framework for Evaluation

**DOI:** 10.5811/westjem.18316

**Published:** 2024-03-29

**Authors:** Elisabeth Fassas, Kyle Fischer, Stephen Schenkel, John David Gatz, Daniel B. Gingold

**Affiliations:** University of Maryland School of Medicine, Baltimore, Maryland

## Abstract

Emergency departments (ED) in the United States serve a dual role in public health: a portal of entry to the health system and a safety net for the community at large. Public health officials often target the ED for public health interventions due to the perception that it is uniquely able to reach underserved populations. However, under time and resource constraints, emergency physicians and public health officials must make calculated decisions in choosing which interventions in their local context could provide maximal impact to achieve public health benefit. We identify how decisions regarding public health interventions are affected by considerations of cost, time, and available personnel, and further consider the role of local community needs, health department goals, and political environment. We describe a sample of ED-based public health interventions and demonstrate how to use a proposed framework to assess interventions. We posit a series of questions and variables to consider: local disease prevalence; ability of the ED to perform the intervention; relative efficacy of the ED vs community partnerships as the primary intervention location; and expected outcomes. In using this framework, clinicians should be empowered to improve the public health in their communities.

## INTRODUCTION

Emergency departments (EDs) in the United States serve a dual role in public health: a portal of entry to the health system, and a safety net for the community at large. Clinically, its position is clear; the ED provides unscheduled acute care, regardless of a patient’s ability to pay. Given its function as a safety net for people lacking consistent access to care, however, the ED is often identified for potential public health interventions due to a perception that it has a unique ability to reach underserved populations. Unsurprisingly, the field of emergency medicine (EM) has taken on this challenge and pioneered a number of effective public health interventions, ranging from community violence prevention^1^ to treatment of opioid use disorder.^2^

One study^3^ identified 43 conditions proposed in the peer-reviewed literature for ED-based public health screening and/or intervention. Given the logistical improbability of any department employing all proposed interventions, clinicians must make calculated decisions about *which* interventions to deploy and *how* to implement them successfully. Unfortunately, there is a lack of evidence-based guidance in the EM literature on how EDs should prioritize and implement such interventions so as to maximally benefit the public health of their local community. These decisions are increasingly important given the growing stress and demands already placed on EDs around the country. Annual patient volumes have increased substantially. Patient acuity is getting more complex. Emergency department boarding has become a national crisis.^4^ Given the significant resource limitations of the ED from these types of factors, any public health intervention beyond core clinical care must have a clear role in the ED setting.

In this paper, we propose a framework grounded in implementation science principles for EDs to prioritize interventions that maximize public health benefits and review the key elements of successful implementation. We present this from the perspective of our own expertise: ED medical directors who have implemented numerous ED-based public health interventions; an emergency medical services medical director working on population health projects; public health researchers and advocates; public policy experts; and emergency physicians. We recognize that the conversation regarding ED-based public health interventions is challenging and affected by many considerations both internal and external to the ED, but we believe success is possible with the right approach.

## PROPOSAL

The volume of potential public health proposals necessitates a framework for determining which are most meaningfully deployed as interventions in a specific ED. As each additional public health screening or intervention takes time within the context of an ED visit, there is a tangible cost to the individual patient associated with participation in public health-focused interventions. Prioritization is challenging for ED administrators, as proposed initiatives rarely arise by a fixed process but rather from a constellation of factors: acute public health emergencies; issues of long-standing concern with individual interest or expertise from a frequently changing physician and nursing staff; strategic initiatives from hospital systems; and often changing priorities from local public health departments or political leaders. Considerations of funding, time, and capacity to provide the intervention with fidelity are often incomplete. Moreover, interventions may be implemented without a plan for rigorous evaluation to justify their continued presence. Given these challenges, a systematic approach to decision-making may maximize health outcomes.

In this context, we provide a framework for considering the merits of conducting a particular intervention within an ED visit. As no ideal framework yet exists, we have adapted constructs from the Consolidated Framework for Implementation Research (CFIR). This implementation framework was originally published in 2009,^7^ representing the cumulative knowledge of implementation science at the time. It is a “pragmatic structure” for effective implementation of programs and systems change—precisely what is needed for enacting effective public health programs in the ED. We did not find all constructs of CFIR pertinent to determining the appropriateness of a new, ED-based public health intervention. Those constructs deemed most relevant, by author consensus, are outlined in Table 1 as a modified framework for considering the merits of a potential intervention. The framework we present is thus a commentary, based on our experience in EM and public health administration.

**Table 1.** Recommended considerations for implementing new emergency department-based public health interventions (Consolidated Framework for Implementation Research model).

CFIR major domains	Relevant CFIR constructs	Questions to consider
Intervention characteristics	1. Evidence strength and quality	• Has the proposed intervention shown effectiveness in patient-centered outcomes in the ED setting? • If not, has the intervention shown benefit that is likely to translate to the ED setting? • How strong is the evidence base?
	2. Relative advantage	• Are there locations other than an ED, either in the hospital or in the community that may be a more patient-centric intervention site? • Can any of these locations perform this intervention more easily, efficiently, cheaply, or effectively?
	3. Adaptability	• Will the local context require any deviations from the established program model? If so, how could these differences impact efficacy? • Does the proposed intervention have the flexibility to evolve, as necessary, after initial implementation?
	4. Trialability	• What is the timeline of the intervention? Is there a clear endpoint? • Will it be possible to end the intervention if not effective?
	5. Complexity	• What challenges might arise to maintaining fidelity to the established program model? • What are possible unintended adverse effects of the intervention for non-participants? Are costs shared, or are specific populations disproportionately harmed? • Are there health equity considerations?
External context^*^	1. Patient needs and resources	• What is the local prevalence of the targeted condition in the general population? The ED population? • Is the targeted population most readily accessible within the ED? Are there alternative and potentially more patient-centered locations? • How does the condition affect local ED utilization, including return visits and hospitalization?
	2. External networking	• Are there effective systems in place to continue care after ED discharge? • How might the absence, change, or loss of external partners affect the intervention?
	3. Peer pressure	• How does the engagement of others in the area affect the need for the intervention and the potential for efficacy?
	4. External policy and incentives	• What stakeholders or policy makers are encouraging implementation? • For programs relying on external funding, what is the long-term stability of this funding?
Organizational characteristics^**^	1. Culture	• Does the intervention fit within the organizational mission of the ED? • Does the intervention fit within the organizational mission of the hospital?
	2. Compatibility	• How does this intervention fit within the existing workflow of the ED? • How would the intervention alter ED performance metrics?
	3. Relative priority	• What essential ED processes might be impacted by the intervention? For example, will throughput be reduced, wait times increased, or triage burdened? • What other programs may need to be sacrificed for implementation? • Do expected benefits outweigh potential disruption?
	4. Leadership engagement	• Is there buy-in from both ED and hospital leadership? • Is there bandwidth within the ED leadership for the intervention?
	5. Available resources	• Will additional resources be required to accomplish the intervention in the ED? How might those resources be made available? • Are there additional outside resources that that could be brought to bear?
	6. Access to knowledge and information	• Is this a condition in which emergency clinicians have specific expertise? • What sources of public health expertise can be tapped within the department? • What additional training or technical expertise might be accessed?
Characteristics of individuals involved	1. Knowledge & beliefs about the intervention	• Are the assumptions supporting implementation in the ED valid?
	2. Individual stage of change	• Are front-line staff motivated to participate in the intervention?
	3. Other personal attributes	• What cultural, religious, or political concerns may staff have about the intervention?
Process of Implementation	1. Planning	• How will the plan be developed and disseminated? • How much time is needed to develop an implementation plan and formulate alliances?
	2. Opinion leaders	• What support or opposition will implementation have from opinion leaders?
	3. Champions	• How is a project champion going to be identified? • Would that champion have the bandwidth, expertise, and influence to overcome obstacles to the intervention?
	4. Executing	• What is the process for continued monitoring and improvement?
	5. Reflecting and evaluating	• What will be the process for evaluation of intervention effectiveness?

* The original CFIR model wording called external setting “outer setting.” The language was changed for clarity when we adapted the framework.

** The original CFIR model called organizational characteristics “inner setting.” The language was changed for clarity when we adapted the framework.

*CFIR*, Consolidated Framework for Implementation Research; *ED*, emergency department.

The CFIR groups implementation science constructs across five domains (intervention, process, individuals, inner setting, outer setting) that can assist systematic assessment of opportunities and barriers to successful implementation. Many of these are well suited to be considered even earlier in the implementation process, as an initial assessment of value and appropriateness. These are posed as priority questions in Table 1. We further explore this proposed framework by discussing its application to several established and experimental, ED-based interventions. These examples are meant to be representative of benefits and challenges that may accompany the implementation of certain interventions. They are not meant to be comprehensive.

## CASE EXAMPLES

Table 2 lists many (but not all) proposed public health interventions in the ED according to level of acceptance and penetrance. Some interventions have become so engrained in the ED workflow that they no longer are perceived as “public health” interventions. Tetanus vaccines, as well as screening for sexually transmitted infections, fall under this category. Below, we explore the proposed framework using individual interventions as case studies as a guide from which to explore the proposed questions. Each example was selected to highlight major considerations required to deploy and maximize public health benefits, and each varies in the extent to which the intervention is accepted and implemented in EDs throughout the country. We consider the overall disease prevalence and impact of the interventions as it relates to future ED utilization. We explore whether the intervention is typically integrated with, runs parallel to, or is separate from the workflow of an ED visit. Similarly, we examine the ability and appropriateness of performing the interventions by considering both financial costs and requisite resources.

**Table 2.** Selective overview of the current state of emergency department‒based public health interventions.

Level of acceptance	Concept	Select examples	Notes
Established	Accepted interventions that are well-integrated in the ED setting	• Sexually transmitted disease testing • Tetanus vaccination • Blood pressure screening • Smoking and tobacco screening • Intimate partner violence screening	Typically codified by current federal guidelines or recommendations, such as The Joint Commission, The Centers for Medicare and Medicaid Services requirements or reimbursement, US Preventive Services Taskforce recommendations.
Supported	Interventions for which implementation is context dependent based on, for example, local epidemiology, local resources, and community priorities.	• Substance use screening, intervention, and referral to treatment • HIV screening and referral for treatment • Hepatitis A and C screening and referral for treatment • Naloxone provision for substance use and overdose • Buprenorphine initiation in the ED for opioid use disorder • Community violence intervention programs • Depression screening and referral	Potentially widely discussed in the emergency medicine literature, these are typically non-regulated interventions that may be the topics of grants or regional implementation. National guidelines may be supportive but not necessarily within the ED setting.
Experimental	Interventions are discussed or implemented at a small number of select departments, often experimental or otherwise research oriented.	• Hepatitis A vaccination • Early pregnancy linkage to care • Dementia screening • Naloxone provision for all opioid prescriptions • COVID-19 vaccination • Screening for housing insecurity and other health-related social needs	Potentially grant funded, these may also be individual departmental projects or the subjects of trials. Well established guidance within or outside the ED is rare.

*ED*, emergency department; *COVID-19*, coronavirus disease 2019.

### HIV Screening

The US Centers for Disease Control and Prevention endorsed ED-based screening for undiagnosed HIV in 2001,^8^ but these recommendations have not risen to the level of official guidelines or quality metrics. Such programs have the potential to test large populations and may find individuals who do not have access to traditional testing programs.

Multiple studies have examined how to best fit HIV screening into existing ED workflow or develop parallel workflows.^9^ Frequent questions include which patients to test (universal vs symptoms vs risk-based screening); who should initiate screening (counselor or clinician); and the operational needs of such programs.^9^ A recent large, randomized trial comparing universal screening against two types of targeted screening showed similar effectiveness in identifying new cases, but with lower resource expenditure of targeted screening programs.^9^ Research demonstrating that clinician-based testing results in lower screening rates suggests the potential benefit of dedicating additional staffing and funds to such initiatives to maximize effectiveness. Operational challenges may further complicate efforts to establish ED-based HIV screenings, including poor linkage to care,^10^^,^^11^ low willingness to test among marginalized populations,^12^^,^^13^ and lack of cultural competency surrounding testing initiatives.^14^^,^^15^ Factors such as lower HIV incidence, improved community awareness and risk-mitigation, increased testing during routine medical care, fewer regulatory barriers to HIV screening in other locations, more effective anti-retroviral medication, and decreased stigma of the disease may also have changed the benefit of ED-based programs since they were first developed more than 20 years ago.

### Intimate Partner Violence Screening

Intimate partner violence (IPV) refers to “physical violence, sexual violence, stalking and psychological aggression by a current or former partner” and affects an estimated one in four women and one in 10 men nationwide.^16^ Screening for IPV in women of reproductive age may help ameliorate physical and psychologic sequelae.^17^^,^^18^ The US Preventive Services Task Force (USPSTF) provides a Grade B recommendation that “clinicians screen for IPV in women of reproductive age and provide or refer women who screen positive to ongoing support services.”

Screening for physical injury could be readily integrated into an ED’s existing assessment of acute injuries. However, for complaints with less obvious connections to IPV, such as mental health conditions exacerbated by IPV exposure, integration of screening may be harder to define or standardize in the absence of universal screening protocols. In practice, universal screening is often deployed while collecting patient information on a myriad of other variables (eg, past medical history, medication history, suicide screening), and may be prone to “click fatigue,” wherein the screener, tasked with compiling a large amount of data in a short amount of time, is unable to perform the screening questions with the intended fidelity.^19^ Patients also can be fatigued by time spent screening for conditions not related to their chief complaint and may be reluctant to divulge sensitive information in this setting. However, focused screening of high-risk populations may miss patients and is prone to bias.

The existing evidence base cited by the USPSTF includes 30 studies, including three random controlled trials (RCT), which yielded nuanced results highlighting the necessity of both components of screening and robust intervention. As Feltner et al report in their conclusion: “Although available screening tools may reasonably identify women experiencing IPV, trials of IPV screening in adult women did not show a reduction in IPV or improvement in quality of life over 3 to 18 months.”^20^ This highlights the challenge of translating positive screens into positive health outcomes. Practicing clinicians will recognize that intervening to protect victims of IPV is challenging when patients present explicitly with this complaint, let alone when patients may be unwilling or unable to divulge symptoms of abuse. Close relationships with community resources equipped to assist victims of IPV are necessary to ensure effectiveness, which requires substantial and sustained administrative support.

### Community Violence Intervention Programs

Gun violence in the United States remains an intractable public health problem, with 2020 recording 19,384 homicides.^21^ In response, hospitals have implemented hospital violence intervention programs (HVIP) in EDs and wards.^22^ These programs use what is described as a “golden moment” of opportunity when patients are in the hospital to foster close, long-term care relationships between culturally competent violence prevention professionals and patients. This includes the creation of comprehensive needs assessments, delivery of case management services, long-term peer support, mental health services, and addressing social determinants of health as root causes of violence.

Initial studies of HVIPs have demonstrated promising results with decreased injury recidivism and improved intermediate outcomes such as delivery of mental health services.^23^ However, to achieve these outcomes, significant commitment is required by EDs, including buy-in from multiple hospital departments, community partners, and internal program champions. The costs of hiring specially trained staff are significant, as time and expertise to perform this intervention is often outside the typical workload of emergency clinicians. Many programs require an annual budget of greater than $300,000. This funding has historically been challenging, although recent developments allow for reimbursement through the Medicaid program in a minority of states.^24^

### Hepatitis A Vaccination

Hepatitis A virus (HAV) is a vaccine-preventable transmissible infection with the potential for long-term, fatal liver disease. A single vaccine dosage is up to 98% effective at preventing transmission.^25^ Consequently, ED-based HAV vaccination has the potential to limit long-term sequelae in those at highest risk of contracting the illness. Still, the process of identifying these at-risk individuals relies on simple screening questions that are often incorporated into standard history-taking instruments and practices in the emergency context. Storage and provision of vaccines can leverage existing hospital pharmacy and nursing protocols. While at-risk groups, including individuals experiencing homelessness or using intravenous drugs, men who have sex with men, and those who have been incarcerated^26^ frequently receive healthcare in the emergency setting, there are also outpatient clinics and other, non-healthcare entities (eg, homeless shelters, nightclubs, jails, substance use treatment facilities) tailored to serve this population. Targeting these community sites may achieve better penetrance of the intervention for underserved population more quickly at lower cost, given modest enrollment of ED-based programs.^27^

### COVID-19 Vaccine Administration

Vaccination efforts based in the ED were also bolstered by the presumptive view that the ED patient population might not have ready access to vaccination outside the ED,^28^ as well as by a desire on the part of many staff members to take part in a national effort of clear import.^29^ An ED-based vaccination seemed to be an obvious extension of hospital-based vaccination programs. The ED vaccination could leverage resources such as ready access to pharmacy and freezers, a relatively small pool of staff who could be trained to administer vaccines, and cultural competency in offering vaccinations. Absent these considerations was an assessment of resource and vaccine availability in the setting of COVID-19-related staffing shortages. The multiple dosing regimen for COVID-19 added complexity and required a separate workflow within the ED context and required follow-up that was sometimes not possible within the ED setting. Additionally, much was unknown about whether the ED offered vaccination to a new or different population or was redundant to other hospital, state, or local community efforts. With varying disease incidence and increasing vaccination rates, there was likely a short window to realize a modest benefit for the intervention.

## DISCUSSION

Emergency physicians are committed to improving public health outcomes, as evidenced by the 2009 and 2021 Society for Academic Emergency Medicine consensus conferences^30^^,^^31^ and the development of several post-EM fellowships in recent years committed to public health and public policy.^32^ Emergency departments have embraced many public health tasks such as screening, surveillance, and interventions outside the traditional scope of emergency care. With limited time and resources, not all public health projects can be undertaken. To maximize public benefit, care must be taken to select interventions that have the largest impact while maintaining integrity to the ED’s core clinical mission. While emergency physicians take pride in the mantra, “anyone, anything, anytime,” we must recognize that some resources may be better spent outside the walls of the ED. This does not mean abandoning certain patient populations, but rather bringing the skills of emergency physicians beyond the walls of the ED through a variety of creative ways, such as collaborations with public health or nonprofit organizations, leveraging emergency medical services experience and connections to develop mobile integrated health programs,^33^ or deploying the tactics of “street medicine.”

Additionally, emergency physicians should consider not just how the program design affects that single condition but how adaptable the intervention is for a specific department and available resources. Consider a hypothetical intervention that may have 90% sensitivity for universal screening, but only 70% for targeted screening. Depending on the difference in staff time between the two, implementing the lower sensitivity targeted approach may in fact allow the same ED to deploy an intervention for an additional public health concern with the marginal resources needed for universal screening, thus maximizing overall benefit.

Screening programs that collect data but do not provide an intervention in response to positive screens are unlikely to be impactful. We posit that the highest value screening programs have appropriate sensitivity and specificity for their target condition, are cost effective, and are actionable. The value of a screening program should be assessed based on the patient population most in need of this screening, the effectiveness of a possible intervention, and the proposed rationale or relative advantage for doing it in the ED. Additionally, buy-in for an intervention is necessary from stakeholders across multiple levels of the organization: hospital and ED leadership, physicians, nursing, and staff. Failure to obtain support from leadership allocating resources or staff carrying out the intervention can damage morale and limit program efficacy.

Interventions for positive screening results, whether for chronic infectious disease or health-related social needs, may need to be provided outside the ED. Therefore, robust external networks between the ED and outpatient clinics and social services are the most important part of a screening and referral program. Most EDs enthusiastically embrace additional resources to coordinate care for their most vulnerable patients, with or without formalized screening programs. Thus, in the planning process EDs should ensure there is significant buy-in from potential external partners, so that any screening implemented has tangible downstream effects. Many may be public clinics or nonprofit organizations that may themselves be underfunded and understaffed, necessitating external funding that should be equitably distributed between stakeholders. External partners often benefit from a champion point of contact in the ED to advertise, monitor, and coordinate referral pathways.

Patient openness to accepting an intervention in the ED is also an important factor in an ED-based intervention. What expectations do patients bring into the ED? For example, a patient suffering an ankle injury may not want to answer questions about their marital sexual practices or smoking habits while awaiting the results of a radiograph. Such questions may be perceived as irrelevant to the stated reason for the visit, and the patient may find them invasive or alienating.

Identifying a literature base for proposed interventions that shows benefit to patient-centered outcomes (eg, improved blood pressure, reduced mortality), or population-based outcomes (e.g., fewer community overdoses or shootings), is an optimal standard for considering implementation of public health intervention in the ED. Observational studies without well-matched controls are often subject to selection bias and regression to the mean. Rigorous evaluation methodology that isolates the effect of the intervention on meaningful outcomes, such as RCTs, is preferred to identify the most impactful interventions. Ideally, implemented interventions will continue monitoring and evaluation of key metrics to ensure local efficacy. When that evidence is absent, we hope that this framework can inform the decision-making process analogous to the way we make clinical decisions in the absence of robust evidence.

Emergency departments are intimately familiar with the ways in which social needs drive healthcare utilization and outcomes. However, disparities in population-based health outcomes are not driven primarily by lack of quality emergency care, but by disparities in broader social determinants of health. These disparities are unlikely to be ameliorated by a one-time intervention within the ED context. Thus, emergency physicians must consider implementing public health programs not as a one-time isolated intervention but rather as the beginning process of long-term, transformative, structural change of the healthcare and social services systems as a whole.^34^

## CONCLUSION

Emergency clinicians and staff care deeply about the public health of the communities they serve. To maximize public health benefit, emergency physicians face challenging decisions regarding which public health interventions hold the most potential for impact, as well as the way they are deployed. Local dynamics will inform decision-making—the balance of benefits and harms may differ on account of context-specific circumstances. Many proposed interventions could also be implemented effectively in some settings but not in others. Given that there is no “one size fits all” approach, we have proposed a framework grounded in implementation science to assess potential interventions in a systematic manner to maximize public health intervention without detracting from the ED’s core function. It is critical to use a guiding framework to properly evaluate efficiency, feasibility, local context, and cost before deployment of any ED-based public health intervention.
